# 

**Published:** 1890-10

**Authors:** 


### HALL'S JOURNAL of HEALTH

ESTABLISHED 1854

CONTENTS.

Vol. 37. No. 10.

| | PAGE. |
|---|---|
| The Death Penalty | 217 |
| The Science of Hygiene | 219 |
| The Husband's Part | 221 |
| On the Strumous Diseases of Childhood | 222 |
| A Tidy and Healthful Home | 223 |
| Cheerful Talk | 224 |
| Physical Education in Relation to Mental Development | 225 |
| What Exercise Does | 227 |
| For Sea Sickness | 227 |
| Swimming Baths for Children | 228 |
| Home the Place to Play | 229 |
| Amazing Virulence of Germs | 230 |
| To Detect Adulteration of Milk | 231 |
| Cod-Liver Oil Substitute | 231 |
| Home-Made Soap | 232 |
| Care of the Skin | 232 |
| The Art of Prolonging Life | 233 |
| Children's Teeth | 234 |
| All Forms of Life Cellular | 235 |
| Diphtheria in Chewing-Gum | 236 |
| Miscellaneous | 237 |
| Arsenic and Ammonia | 239 |
| Literary | 240 |
| Maiden Fair | 240 |

INDEX TO ADVERTISEMENTS OF HALF A PAGE AND OVER.

| | PAGE. |
|---|---|
| Pearline | I |
| Rochester Lamp Co | II |
| Herba-Vita Remedy Co | III |
| Burnett's Extracts | IV |
| Ayer's Sarsaparilla | V |
| Goldsmith's Pianos | VI |
| Hood's Sarsaparilla | VIII |
| Celluloid | IX |
| Hospital Remedy Co | X |
| Bovinine | XI |
| Hall's Journal of Health | XII |
| R. W. Shoppell | XII |
| Parke, Davis & Co | XIII |
| Castoria | XIII |
| Sapolio | XIV |
| Royal Baking Powder | XV |
| Merriam & Co | XV |

